# Supplementary figures and images for: BNT162b2, mRNA-1273, and Sputnik V Vaccines Induce Comparable Immune Responses on a Par With Severe Course of COVID-19
Source: Front Immunol. 2022 Apr 13;13:797918. doi: 10.3389/fimmu.2022.797918 (PMC9044856; doi:10.3389/fimmu.2022.797918)

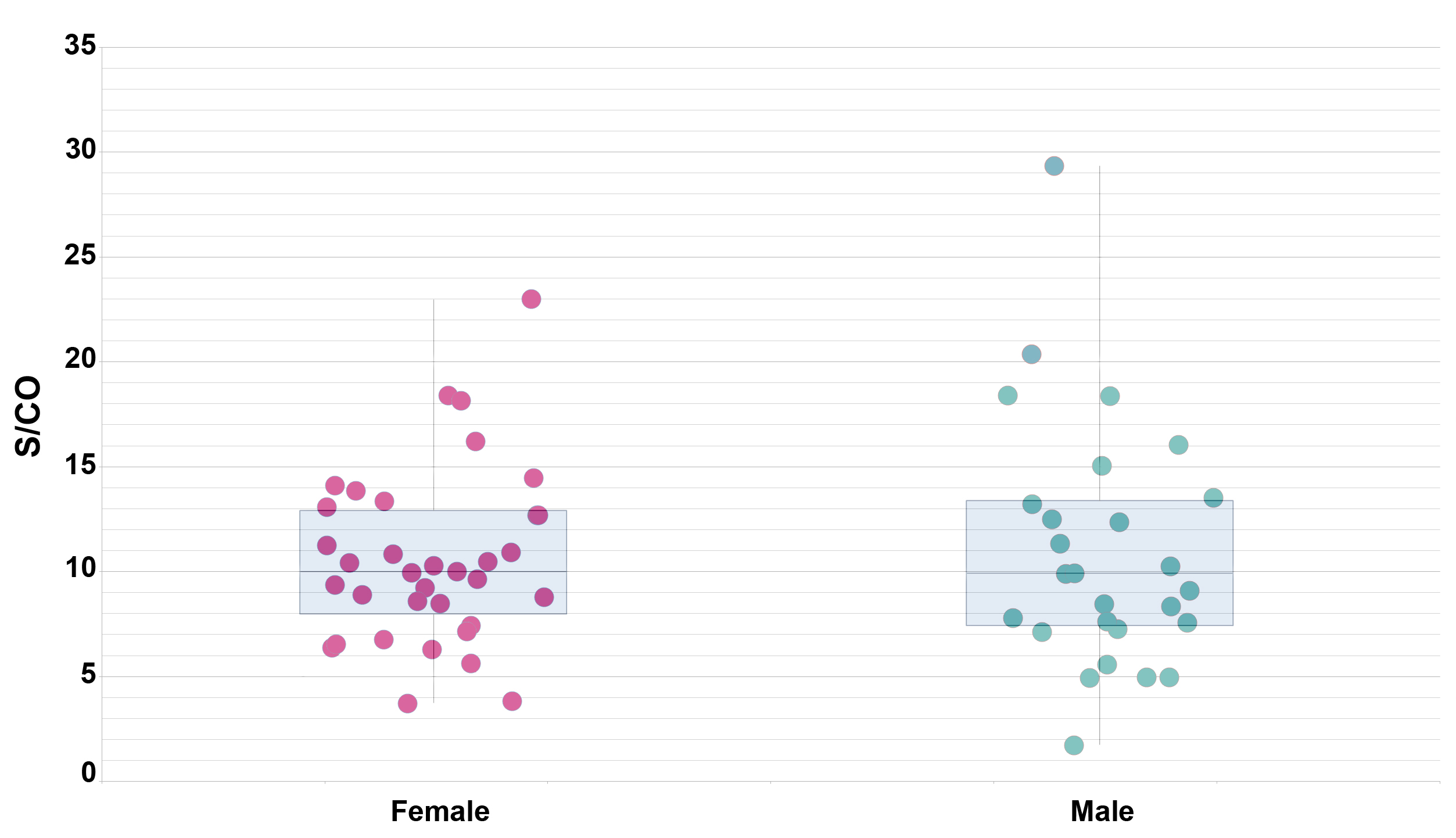

Supplement: Supplementary file 1 [file Image_1.jpeg]
